# Knowledge, attitudes, and practices of pediatricians on infantile colic in the Middle East and North Africa region

**DOI:** 10.1186/s12887-017-0939-0

**Published:** 2017-10-23

**Authors:** Flavia Indrio, Mohamad Miqdady, Fahd Al Aql, Joseph Haddad, Berkouk Karima, Katayoun Khatami, Nehza Mouane, Aiman Rahmani, Sulaiman Alsaad, Mohamed Salah, Gamal Samy, Silvio Tafuri

**Affiliations:** 1 Department of Pediatric University of Bari Ospedale Pediatrico Giovanni XXIII Hospital, Via Amendola 270, 70126 Bari, Italy; 2 Hepatology & Nutrition Division, Pediatric Gastroenterology, Sheikh Khalifa Medical City, Abu Dhabi, United Arab Emirates; 3 King Fahad Medical City, Riyadh, Kingdom of Saudi Arabia; 4 Department of Pediatrics, Saint George University Hospital, Balamand University, Beirut, Lebanon; 5 Department of Pediatrics, Bab El Oued Hospital, Algiers, Algeria; 6 Department of Pediatric Gastroenterology, Hepatology and Nutrition, Mofid Children Hospital, Shahid Beheshti University of Medical Sciences, Tehran, Iran; 7 Gastroenterology Nutrition Department, Children Hospital Ibn Sina, University Mohammed V Faculty of Medicine, Rabat, Morocco; 8 Tawam Hospital, Al Ain, United Arab Emirates; 9 Royale Hayat Hospital, Kuwait City, Kuwait; 10 Nestlé Nutrition, Dubai, United Arab Emirates; 11 Department of Child Health and Nutrition, Institute of Postgraduate Childhood Studies, Ain Shams University, Cairo, Egypt

**Keywords:** Colic, Functional gastrointestinal disorder, Middle East and North Africa, Pediatricians, Breastfeeding, Formula, Infant, Fussing, Neonate

## Abstract

**Background:**

Regional evidence-based guidelines for the prophylaxis and management of infantile colic are not available for the Middle East and North Africa (MENA) region. The Allied Against Infantile Functional GI Disorders (ACT) Working Group was created in January, 2015 to determine the knowledge gaps and the current management practices of infantile colic by physicians in the MENA region. The ACT group determined the need for a survey to address these questions. The objectives of the survey were to highlight current clinical practices on the management of infantile colic and to raise awareness on colic severity in the MENA region.

**Methods:**

The ACT working group developed the survey which included respondent characteristics and closed questions on practice in colic prevention. The survey was subject to validation and ethics committee approval in all countries.

**Results:**

A total of 1628 physicians (mostly pediatricians (75.4%), neonatologists (2.4%) and general practitioners (19.8%)) responded to the survey. The 5 most represented countries were KSA (27.9%), Kuwait (22.1%), Morocco (13.8%), Lebanon (10.6%), and Iraq (7.4%). Most of the respondents (77.8%) practiced in governmental settings. A majority of respondents (91.7%) reported that colic is diagnosed predominantly by clinical examination. Above 63%, of pediatricians surveyed, believed that the colic prevalence rate was >40%, which is greater than the 20% rate reported in worldwide surveys. Yet, most of the responding physicians (73%) prefer to simply reassure parents rather than prescribe a therapeutic agent. Most physicians were either neutral (58%) or did not endorse (18.4%) colic prophylaxis. Of those who prescribed formulae for non-breastfed children, a majority (64.3%) chose “Comfort” formulae over hydrolyzed or lactose-free formulae or formulae with probiotics.

**Conclusions:**

The results of this survey suggest that a substantial proportion of responding physicians from the selected MENA countries do not advocate for prophylaxis of colic. The findings of this survey suggest that more educational efforts are required to increase awareness of the strong body of evidence supporting the efficacy of probiotics in the prevention and management of infantile colic.

**Electronic supplementary material:**

The online version of this article (10.1186/s12887-017-0939-0) contains supplementary material, which is available to authorized users.

## Background

Functional gastrointestinal disorders (FGIDs) are defined as a variable combination of chronic or recurrent gastrointestinal symptoms not explained by organic abnormalities. The exact pathophysiology underlying these disorders is unclear and several factors are thought to be involved in their expression. FGIDs in childhood are age dependent, and the Rome Foundation has established two pediatric committees to identify the criteria for diagnosis of FGIDs: the Infant/Toddler (up to 4 years) Committee and the Child/Adolescent Committee (aged 4–18 years) [1]. During infancy, infantile colic and gastroesophageal reflux are probably the most common FGIDs that lead to referral to a pediatric gastroenterologist [2, 3].

Infantile colic, as per the classical Wessel’s definition, appears at a very early age in otherwise healthy infants who experience unexplained and inconsolable crying episodes lasting for more than 3 h per day, for 3 or more days per week, and for 3 or more weeks (for at least 1 week in Rome III Criteria). Crying episodes, which usually peak around 6–8 weeks and gradually resolve spontaneously by 3–4 months of age, are accompanied by painful expression, flushing, flexing of the hips, and distended abdomen with flatulence. The precise etiology of colic is still unknown, but food allergy and gut function immaturity and dysmotility are thought to have some causative contribution [4–6]. Although not considered a serious problem by many pediatricians, infantile colic is the cause of 10–20% of all pediatrician visits in the first 4 months of life, and can lead to excessive parental anxiety, exhaustion, and stress [7]. Although a wide range of infant colic prevalence (2–73%) has been reported, experts generally agree on a 20% prevalence rate worldwide [7]. Furthermore, there is evidence of intestinal neutrophilic infiltration and different microbiota in colicky infants compared with non-colicky infants, resulting in low-grade intestinal inflammation that may lead to gastrointestinal disorders reported later in life [8–10].

Although the diagnostic criteria for infantile colic are clearly stated in the Rome III Criteria [1], one standard criteria has not been universally accepted for the management of diagnosis and therapy. Currently, parents and pediatricians use several therapeutic approaches such as reassurance of parents, use of partially hydrolyzed protein formula, use of low-lactose formula, change of infant formula, interruption of breastfeeding, and use of herbal or other naturalistic products. Frequently these options, which are not all evidence based, can be dangerous and may have side-effects. Moreover, they are not effective and reassurance may not be enough for anxious parents who may seek a second opinion from other physicians, family members, or online advice.

There is growing evidence that infantile colic may be associated with a different pattern of intestinal microbiota compared with healthy controls [11]. Molecular methods to evaluate the gastrointestinal flora colonization patterns in infants with colic have identified an increase in intestinal coliform bacteria, particularly *Escherichia coli* [12]. Phylogenetic microarray analysis determined that colicky infants displayed lower microbiota diversity and stability than control infants [13]. Furthermore, infants with colic presented with more than double the level of proteobacteria, but reduced levels of bifidobacteria and lactobacilli [13]. A separate study suggests that administration of bifidobacteria and lactobacilli appears to protect against crying and fussing [14]. Consistent with this growing body of evidence, probiotics are rapidly emerging as a valuable therapeutic option that confer health benefits in the treatment and prevention of infantile colic [15]. Probiotics colonize the bowel, where they function to strengthen mucosal barriers, prevent other bacterial colonization, inhibit intestinal inflammation, and regulate the development of infant gut microbiota [16, 17].

To date, no investigation into the incidence and management of infantile colic in the Middle East and North Africa (MENA) region have been performed. The aim of this paper is to determine the perceived regional incidence of colic and to assess the main diagnostic and therapeutic procedures used for this condition. A secondary aim of the paper is to assess the perceived value of probiotics in the management of infantile colic.

## Methods

### Survey design

An anonymous questionnaire survey (see Additional file 1: Appendix) was developed by a working group of pediatrician experts with reference to existing research literature. The working group comprised regional experts from representative countries across the MENA region including Egypt, Kingdom of Saudi Arabia, Kuwait, Lebanon, Morocco and the United Arab Emirates. International experts from Italy consulted on the design of the survey. The Nestlé Nutrition Institute Middle East also collaborated with this initiative during a meeting in Dubai in January 2015.

The survey was structured into 15 items on diagnosis and treatment of infantile colic:

1. Specialty of the enrolled physician
2. Setting where the interviewee worked (government or private facility; clinical/hospital/other)
3. Country
4. City
5. Gender
6. Age group (<40 years; 40–50 years; 51–60 years; >60 years)
7. Full-time/part-time worker
8. The percentage of infants with gastrointestinal complaints among infants aged 0–4 months
9. The percentage of infants (0–4 months of age) who suffered from colic
10. Risk factors for infantile colic (male gender, prematurity, formula feeding, first born baby, family distress)
11. The symptoms most frequently associated with infantile colic
12. The tools used by the physician to diagnose colic
13. The attitude of parents when the physician seek their advice
14. The treatment measure considered by the enrolled physician, and when it was deemed necessary to change the formula (e.g. to prescribe ‘Comfort’ formula, formula with probiotics, hydrolyzed formula, or lactose-free formula)
15. The perception of prophylaxis against infantile colic.

In question 14, ‘Comfort’ formula indicates a partially hydrolyzed protein, low in or free from lactose and containing a modified fat blend. ‘Hydrolyzed formula’ indicates hydrolyzed protein containing formula.

The survey was completed by pediatricians, general physicians, and neonatologists predominantly in the Kingdom of Saudi Arabia (KSA), Kuwait, Morocco, Lebanon, Iraq, Algeria, Egypt, Iran, United Arab Emirates (UAE), Jordan, Palestine, and Oman. Because the number of participants from Jordan, Oman, and Palestine was less than 20, in the results they were grouped as ‘other’.

### Blinding and statistical analysis

The questionnaire was validated by a group of 15 pediatricians. When consensus was reached, the authors distributed the questionnaire to 1800 practicing healthcare professionals to attendees of national and regional general pediatric meetings in participating countries from the MENA region. The questionnaires were completed by 1628 physicians anonymously. Convenience sampling was employed to collect data. To preserve blinding, only personnel exclusively designated for recording data evaluated the responses. Blinded data (entered by two different people) were entered into a Google Drive platform database and analyzed with the STATA MP11 statistical software. Results were described as percentages with 95% confidence intervals (CIs), where appropriate. The authors met in January 2015 to discuss the data and to reach a consensus on the knowledge base and practice trends towards infant colic in the MENA region.

## Results

A total of 1628 doctors (57.6% male, 42.4% female) completed the questionnaire, of which 75.4% (*n* = 1227) were pediatricians, 19.8% (*n* = 323) were general practitioners, 2.4% (*n* = 39) were neonatologist, and 2.4% (*n* = 3.9) were other healthcare workers. A total of 77.8% (*n* = 1266) worked in a government facility and 22.2% (*n* = 362) worked in a private facility; 67.6% (*n* = 1101) were employed in a hospital and the remaining in a clinical setting. A total of 89.9% (*n* = 1463) worked full time. Distribution of enrolled people per country is reported in Table 1.

**Table 1:** Distribution of enrolled physicians per country

Country	n	%
KSA	455	27,9
Kuwait	360	22,1
Morocco	224	13,8
Lebanon	172	10,6
Iraq	120	7,4
Algeria	88	5,4
Iran	66	4,1
Egypt	68	4,2
UAE	40	2,5
Other	35	2,1
Total	1628	100,0

**Fig. 1:**
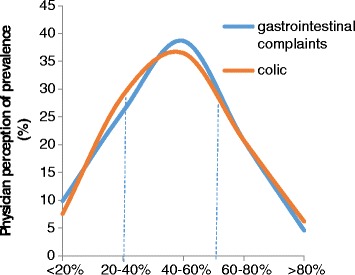
Estimated prevalence of gastrointestinal complaints and colic among subjects aged 1–4 years old

Figure 1 shows the perception of enrolled pediatricians on the prevalence of common conditions of intestinal sensitivity. All healthcare professionals reported similar perception of prevalence rates for colic and other gastrointestinal complaints. More than 63% of physicians across all countries, represented in this survey, believed that the colic prevalence rate in infants below the age of 4 months was higher than 40%, which is consistent with the rate of all other gastrointestinal complaints.

The estimated prevalence of gastrointestinal conditions and colic in infants for each country is reported in Table 2. These results reveal that although pediatricians in Algeria and Egypt tend to perceive different numerical trends for gastrointestinal conditions and colic prevalence rates, most of the pediatricians in the other countries felt that gastrointestinal conditions and colic prevalence closely mimicked each other. Prevalence of gastrointestinal complaints and infantile colic was numerically lower in infants from Iran and higher in infants from Algeria (Table 2).

**Table 2:** Estimated prevalence of gastrointestinal complaints and colic among subjects 1–4 years old, per country

Country	<20%		20–40%		40–60%		60–80%		>80%	
	GIC	IC	GIC	IC	GIC	IC	GIC	IC	GIC	IC
Algeria	5,7	6,8	14,8	14,8	47,7	29,5	25,0	33,0	6,8	15,9
Egypt	10,3	1,5	30,9	25,0	33,8	30,9	20,6	23,5	4,4	19,1
Iran	24,2	19,7	34,8	39,4	25,8	28,8	12,1	9,1	3,0	3,0
Iraq	6,7	5,8	27,5	30,8	40,0	30,0	19,2	23,3	6,7	10,0
KSA	15,2	9,7	29,5	30,5	34,5	37,4	17,6	18,0	3,3	4,4
Kuwait	4,2	3,9	19,2	29,7	45,8	41,7	26,4	21,4	4,4	3,3
Lebanon	8,7	7,0	35,5	36,0	39,5	35,5	13,4	16,9	2,9	4,7
Morocco	6,7	6,3	24,1	22,8	34,8	37,5	29,5	27,7	4,9	5,8
UAE	15,0	15,0	15,0	25,0	52,5	35,0	7,5	17,5	10,0	7,5
Other	17,1	17,1	28,6	25,7	31,4	37,1	11,4	8,6	11,4	11,4
Total	10,0	7,6	26,0	28,9	38,7	36,5	20,8	20,8	4,5	6,2

*GIC* gastrointestinal complaint; *IC* infantile colic

A total of 37% (*n* = 604; 95% CI 34.7–39.4) of enrolled pediatricians stated that formula feeding was the most important risk factor for infantile colic, and 29.3% of enrolled pediatricians (*n* = 476; 95% CI 27–31.4) indicated prematurity as a major determinant. Almost a quarter of pediatricians (23.4%; *n* = 381; 95% CI 21.3–25.5) considered male gender to be the main risk factor; 6.4% (*n* = 104; 95% CI 5.2–7.6) believed being the first-born baby was a risk factor, and 3.9% (*n* = 64; 95% CI 3–4.9) reported family distress as a risk factor. A very low percentage of interviewed pediatricians from Algeria (7.9%) indicated male gender as a risk factor, whereas only 12.1% of pediatricians from Iran indicated prematurity as a risk factor. Pediatricians from Algeria (10.2%) and Iran (13.6%) also seemed more likely to consider family distress as a risk factor.

The symptoms more frequently associated with infantile colic were abdominal distension (*n* = 1093; 67.1; 95% CI 64.5–69.4), feeding disorders (*n* = 856; 52.6%; 95% CI 50.1–55.0), sleeping disorders (*n* = 894; 54.9%; 95% CI 52.5–57.3), and abnormal stool consistency (*n* = 618; 38%; 95% CI 35.6–40.3).

A total of 91.7% (*n* = 1493; 95% CI 90.4–93) of interviewed physicians stated that their diagnosis was based on clinical evaluation; only 4.1% (*n* = 67; 95% CI 3.1–5.1) reported the use of stool test and a small number of doctors used blood tests (2.5%; *n* = 40; 95% CI 1.7–3.2) or radiological imaging (1.7%; *n* = 27; 95% CI 1.0–2.3).

According to 68.2% of respondents (*n* = 1111; 95% CI 66–70.5), parents usually changed formula before seeking advice from a pediatrician; 25.4% (*n* = 414; 95% CI 23.3–27.5) reported that parents used herbal treatment; 3.4% (*n* = 55; 95% CI 2.5–4.3) stopped breastfeeding; 2.2% (*n* = 35; 95% CI 1.4–2.8) used probiotics; and 0.8% (*n* = 13; 95% CI 0.4–1.2) used cautery. The attitude in changing formula was unusual for Egyptian (27.9%) and Iranian (39.4%) pediatricians. According to interviews from these countries, parents were more like to use herbal treatments (69.1% for Egyptian and 54.5% for Iranian parents) than other treatment options.

The majority of interviewed pediatricians (72.8%; *n* = 1182; 95% CI 70.4–74.8) reported reassuring parents as part of standard treatment in cases of infantile colic; 14.3% (*n* = 233; 95% CI 12.6–16) considered changing formula, 4.8% (*n* = 79; 95% CI 3.8–5.9) considered herbal treatment, 4.5% (*n* = 73; 95% CI 3.5–5.5) considered probiotics, and 3.8% (*n* = 61; 95% CI 2.8–4.7) considered simethicone. Changing formula was more frequent among pediatricians from KSA (22.2%) and Kuwait (20.3%).

Table 3 shows the attitudes of pediatricians in changing formula, when appropriate, for a non-breastfed baby. Pediatricians from Iran (40.9%) were less likely to prescribe ‘comfort’ formula and preferred hydrolyzed formula (25.8%). Pediatricians from Egypt (25%) and Iran (12.1%) were most inclined to prescribe lactose-free formula. Only 23.4% (*n* = 384; 95% CI 21.5–25.6) endorsed the concept of prophylaxis against infantile colic whereas 18.4% (*n* = 300; 95% CI 16.5–20.3) did not, and 58% (*n* = 944; 95% CI 55.6–60.4) stated they had no opinion.

**Table 3:** Distribution of enrolled pediatricians by recommendation of when formula must be changed for a non-breastfed baby

Recommendation	n	%	95% CI
‘Comfort’ formula	1047	64.3	62–66.6
Formula with probiotics	261	16	14.2–17.8
Hydrolyzed formula	198	12.2	10.6–13.8
Lactose-free formulas	109	6.7	5.5–7.9
Other	13	0.8	0.4–1.2
Total	1628	100	

## Discussion

The pathogenesis underlying FGID of the infant remains elusive, and no evidence-based form of therapy has been widely adopted thus far. Parental education, reassurance, and anticipatory guidance are still recommended as first-line approaches in the management of FGID in infants, and medications are usually not indicated. The prevalence of FGIDs, specifically infantile colic, in the MENA region appears to be much higher than the 20% rate reported in worldwide surveys [7]. These data confirm reports in the literature that this increase in rate is not related to race, social, or cultural differences [7]. The diagnosis was performed in most cases using the clinical definition from the Rome III Criteria. The associated symptoms reported were feeding difficulties associated with abdominal distension and sleeping disorders [18]. These symptoms are the same as those reported in literature in other parts of the world [4, 5].

This is the first survey on pediatrician and general practitioner knowledge of and attitude towards infantile colic in the MENA region. Although a significant number of papers on infantile colic have been published for more than 45 years, there is no adequate consensus on the most efficient way to treat these patients and, generally, the interventions are selected based on experience rather than on evidence. Evidence-based analysis using traditional approaches and single meta-analysis have demonstrated conflicting results when the different therapeutic options for colic have been evaluated [15, 19–23].

Although the increased rates of colic reported by pediatricians were largely acknowledged to be closely relate to gastrointestinal complaints, very few pediatricians advocated gastrointestinal remedies. The predominant approach used by pediatricians is parental reassurance. Although this is consistent with worldwide practices [24], given the higher than norm prevalence and the predominant tendency of parents in this region to either change formula or try ineffective herbal medications, most pediatricians did not seem to be counseling parents towards more corrective measures, such as probiotics. The high rate of physicians reporting a neutral attitude towards infantile colic prophylaxis reflect this lack of urgency.

The persistent crying and discomfort suffered by infants may adversely affect the quality of life of parents, with reports of increased maternal depression and a general deterioration of parents’ psychological status [25]. Considering the favorable clinical course of infantile colic, conservative treatments strategies, such as avoiding overfeeding, should be adopted in the appropriate clinical setting. Non-analgesic, non-nutritive soothing maneuvers, such as rhythmic rocking and patting 2–3 times per second in a quiet environment, may temporarily soothe a baby who may resume crying when placed in their cot. Rhythmic motion is a common maneuver that does not eliminate pain but may stop crying (e.g. a car ride); however, although this has diagnostic and therapeutic value, it should not be overdone. Other harmful practices like cautery, which is still prevalent in some countries, must be stopped. A study of 150 age- and gender-matched infants in Saudi Arabia revealed that 14% of these infants underwent cautery, performed by a traditional healer, because of excessive crying [26]. Assessments should measure parents’ coping skills and anxiety level to prevent potential child abuse in the form of shaking baby syndrome. Management consists of helping parents cope, and any measure that parents perceive as helpful is worth continuing provided it does not cause harm.

The most frequent parental responses to colic management are to change the infant formula (68%) prematurely and to stop breastfeeding (3%). Major changes in feeding can result in changes in the microbiota, which may eventually disrupt the balance of inflammation in the intestinal mucosa. This practice should be avoided as much as possible. Other therapies investigated for the treatment of infantile colic are simethicone, cimetropium bromide, dicycloverine, trimebutine, and proton pump inhibitors. However, very few have shown clinically meaningful benefit [24]. An alternative to completely switching diets is to temporarily add formula containing probiotics to the baby’s existing diet to help normalize the gut microbiota while maintaining consistency in nutrition.

In the past 5 years, this novel therapeutic approach has been increasingly used by pediatricians. The use of certain probiotics in the treatment of colic relies on several factors [15, 19–22, 27–39]. The enteric microbiota can influence gut motility, visceral sensitivity, abnormal brain–gut interaction, and immune responses [2, 8, 9, 40–43]. These factors have all been suggested as crucial for the development of FGIDs, and the manipulation of microbiota through pre/probiotic supplementation is an important and expanding field in the prevention and management of these diseases [20, 22, 24, 30–34, 38, 44, 45]. To date, two high-quality meta-analyses are available in the management of infantile colic by means of probiotics, and *Lactobacillus reuteri*, which is found in breast milk, seems to be an effective treatment for crying in exclusively breastfed infants with colic [15, 39]. An improvement in gut function, motility, and visceral pain has been suggested as a few of the benefits of *Lactobacillus reuteri* administration. Reduced levels of *E. coli* were also observed, leading some to speculate that the improvement in colic symptoms could be partly due to changes in fecal microbiota [11]. Although parental reassurance should still be the primary treatment measure for infantile colic, the growing robust evidence on the effectiveness of supplemental probiotics in this condition should be considered to provide adjuvant therapeutic relief to these infants.

It is important to note that not all probiotics can be used for this indication. In the MENA region, probiotics are only used for the treatment of infantile colic in 4.5% of cases. This is possibly because 50% of doctors were unsure what preventative methods to recommend.

Recently, Indrio et al. demonstrated that preventive intervention in infants not only reduces the probability of colic episodes, but also reduces the number of pediatric visits or visits to the emergency department due to digestive symptoms, the parent’s absenteeism, and the use of non-approved intervention such as simethicone or herbal products [34, 38]. Subsequently, the cost to the family and community in the treatment of colicky infants was also impacted, with a mean saving of $118.71 for the family and $140.30 for the community per patient [34]. These savings may not occur with simethicone because studies have demonstrated simethicone’s relatively poorer efficacy in treating colic in infants compared with probiotics [46, 47].

### Limitations

By its nature, the survey method and the convenience sampling method is limited by its non-random method of participant selection. The survey only collected information on healthcare practitioners’ self-reported management of infantile colic. No attempts were made to determine actual local clinical practice. The diverse healthcare systems in the different countries could also impact the practice patterns of physicians. For example, the access and availability to certain therapies could shape treatment optimization strategies.

## Conclusion

The higher prevalence rates of infant colic reported by physicians in the MENA region compared with those reported worldwide is indicative of the urgent need for more active preventative measures than those currently advocated by international guidelines. The traditional approach of parental reassurance does not adequately assuage the worries of the parents, which could lead to the use of alternative erroneous approaches suggested by family, friends, or the internet. Some of these options have not been vetted by scientifically sound studies and may be harmful (e.g. cautery to the abdomen). Thus far, the new strong body of evidence supporting the efficacy of probiotics in the prophylaxis of infantile colic has not been incorporated in the guidelines and should be taken into consideration when counseling parents. In light of this recent evidence, preventive treatment, such as the use of probiotic *Lactobacillus reuteri*, seems to be promising and may have an individual and societal cost benefit. Combining probiotic use with parental reassurance may therefore be advisable pending larger scale confirmatory studies of the positive benefits of *L. reuteri* on the prevention and treatment of colic [8, 20, 27, 34, 35, 38, 42, 43].

## Additional file

Additional file 1: Appendix. Allied Against Infantile Functional GI Disorders (ACT) Infantile Colic Survey. (DOCX 19 kb)

## Abbreviations

CI: Confidence interval
FGID: Functional gastrointestinal disorder
MENA: Middle East and North Africa
UAE: United Arab Emirates
