# Supplementary material for: Knowledge, attitudes, and practices of pediatricians on infantile colic in the Middle East and North Africa region
Source: BMC Pediatr. 2017 Oct 23;17:187. doi: 10.1186/s12887-017-0939-0 (PMC5651634; doi:10.1186/s12887-017-0939-0)
Supplement: Additional file 1: — Appendix. Allied Against Infantile Functional GI Disorders (ACT) Infantile Colic Survey. (DOCX 19 kb) [file 12887_2017_939_MOESM1_ESM.docx]

**Additional file 1: Appendix**

**Allied Against Infantile Functional GI Disorders (ACT) Infantile Colic Survey**

Dear colleague,

Greetings.

In an effort to better understand infantile colic in the region; we would appreciate your response to the below questionnaire. Estimated required time: 3-5 minutes.

1. Specialty
2. Setting where the interviewee worked (government or private facility; clinical/hospital/other)
3. City, Country
4. Gender
5. Age group (<40 years; 40–50 years; 51–60 years; >60 years)
6. Full-time/part-time worker
7. In your practice; what is the percentage of infants with gastrointestinal complaints among all infants age 0-4 months?
   - 1. <20%
     2. 20-40%
     3. 40-60%
     4. 60-80%
     5. >80%
8. What is the estimated percentage of infants who suffer from colic (0-4 months of age)?
   - 1. <20%
     2. 20-40%
     3. 40-60%
     4. 60-80%
     5. >80%
9. What are the risk factors for infantile colic? (may have more than one answer)
   1. Male gender
   2. Prematurity
   3. Formula feeding
   4. First born baby
   5. Family stress
10. Which symptoms associated you see more frequently? (may have more than one answer)
    1. Feeding disorders
    2. Abnormal stool consistency
    3. Abdominal distension
    4. Sleeping disorders
11. What tools do you use to diagnose?
    1. Clinical
    2. Blood test
    3. Radiological imaging
    4. Stool test
    5. Others: please specify ………
12. What do parents usually do before they seek your advice?
    1. Formula change
    2. Stop breast feeding
    3. Herbal treatment
    4. Probiotics
    5. Cautery
13. What treatment measure do you consider?
    1. Reassurance of parents
    2. Formula change
    3. Herbal treatment
    4. Probiotics
    5. Simethicone
14. In the non-breast fed baby; if formula to be changed; what do you recommend?
    1. “Comfort” formula
    2. Hydrolyzed formula
    3. Formula with probiotics
    4. Lactose free formulas
    5. Others: please specify ………..
15. What is your perception of the concept of “Prophylaxis” against infantile colic?
    1. Endorse
    2. Neutral
    3. Don’t endorse
